# A comparative study of the effects of Kangaroo care by mothers and maternal grandmothers on the vital signs of hospitalized preterm newborns: a randomized controlled clinical trial study

**DOI:** 10.1186/s13063-023-07288-y

**Published:** 2023-04-14

**Authors:** Zahra Dargahiyan, Fatemeh Ghasemi, Kimia Karami, Fatemeh Valizadeh, Rasool Mohammadi

**Affiliations:** 1 School of Nursing and Midwifery, Lorestan University of Medical Sciences, Khorramabad, Iran; 2 Social Determinants of Health Research Center, School of Nursing and Midwifery, Lorestan University of Medical Sciences, Khorramabad, Iran; 3 Department of Pediatrics Nursing, School of Nursing and Midwifery, Lorestan University of Medical Sciences, Khorramad, Iran; 4 Department of Biostatistics and Epidemiology, School of Public Health and Nutrition, Lorestan University of Medical Sciences, Khorramabad, Iran

**Keywords:** Preterm newborn, Kangaroo care, Mother, Maternal grandmother, Vital signs

## Abstract

**Background:**

Kangaroo care (KC) is an effective technique to prevent injury in newborns due to prematurity and hospitalization. Mothers of preterm newborns experience their own set of physical and mental problems. Such circumstances call for another family member to take care of the newborn. This study compared the effect of KC by mothers and maternal grandmothers on the vital signs of preterm newborns.

**Methods:**

This parallel randomized controlled trial was done at the neonatal and NICU departments of the hospital in Kuhdasht in Iran. Eighty preterm neonates were selected through convenience sampling according to the eligibility criteria, then by stratified block randomization allocated to two groups. The control group received KC from the mother, and the intervention group received KC from the maternal grandmothers on the vital signs of preterm newborns. Vital signs were assessed 15 min before, during, and after the KC as the primary outcome. The data collection tools included a demographic questionnaire and a form to record the vital signs. Vital signs were measured by a pulse oximeter, an electronic thermometer, and observation. Data were analyzed by the chi-square test, the independent *t*-test, and the repeated measures ANOVA.

**Results:**

The vital signs of newborns in each group showed a significant difference before, during, and after receiving KC (*P* < 0.05). Nevertheless, the vital signs of the newborns did not differ significantly between the mother and the maternal grandmother KC groups (*P* > 0.05).

**Conclusion:**

KC by maternal grandmother may stabilize the vital signs of preterm newborns as much as when this type of care is provided by the mother. We, therefore, recommend the provision of KC by the maternal grandmother, as a support and substitute for the mother whenever she is incapable of being at the hospital and to enable the mother to rest.

**Trial registration:**

Iranian Registry of Clinical Trials IRCT20211225053516N1, March 31, 2022.

## Introduction

Prematurity and low birth weight are among the most common problems in newborns [1, 2]. Premature newborns make up approximately 15% of all newborns worldwide, and 70% of all neonatal mortalities occur in this group [3].

In 2019, there were 5.30 million deaths (95% CI: 4.92–5.68) among children younger than 5 years, primarily due to preterm birth complications [4].The prevalence of low birth weight (LBW( in the Iranian population based on a systematic review was a total of 10% (95% CI: 9–12) in 2016 [5] and 7.95% (95% CI: 7.36–8.58) in 2019 [6].

Preterm newborns will face more challenges than healthy newborns as a result of the insufficient growth of their bodily organs. These newborns are unable to tolerate physiological tensions. They experience more physical, mental, and emotional issues such as sensory impairment and cognitive and language impairment compared to full-term newborns [7–9]. They require professional healthcare for normalized growth and survival due to their physiological characteristics, including ineffective respiratory patterns followed by a drop in arterial blood oxygen and the risk of hypothermia [9, 10]. Given these complications, the use of natural and supportive methods of care, such as kangaroo care (KC), in preterm newborns is considered an easy and preventive care method [11]. It is one of the best active care processes to prevent preterm injury and hospitalization [1, 11]. In this type of care, the newborn is placed vertically on the parents’ chest as skin-to-skin contact, increasing diaphragm efficiency, lung function, and oxygen delivery to the newborn. So, it leaves a positive effect on respiration, heart rate, and SpO_2_ [12] and improves the stability of the cardiovascular and respiratory systems. Also, it facilitates primary transition, promotes breastfeeding and weight gain, reduces the length of hospital stay, and consequently reduces the cost of care [1, 11–13].

Nevertheless, mothers of preterm newborns experience their own set of physical and mental problems as a result of their premature delivery. They are sometimes unable to be at the hospital to take care of their newborn due to fatigue, medical conditions, multiple pregnancy, or other reasons, such as surgical incisions [14, 15]. Such circumstances call for another family member to take care of the newborn [14, 16].

A few studies have assessed the participation of other family members apart from the mother, such as the father, in performing this type of care. Nonetheless, these studies have shown that fathers face many challenges during the birth and hospitalization of preterm and low-birth-weight newborns. These are including confusion, stress, anxiety, and financial and occupational issues associated with the newborn’s hospitalization, which play a significant part in their parenting role and are significant factors contributing to their distance from the newborn and the ward [17, 18]. In a study by Valizadeh et al. in Iran, fathers were unwilling to participate directly in taking care of their newborns in the ward due to the traditional belief that taking care of babies is the mothers’ duty [19].

Also, given that Iran is an Islamic country with strict religious beliefs about privacy and values, and also given the culture and beliefs governing the research environment, the presence of fathers is an obstacle to other mothers’ breastfeeding or KC. Private NICU spaces are limited in Iran, and the participation of fathers in taking care of their newborns may create unease for other mothers during breastfeeding [19–21]. Hence, nurses committed to the privacy of mothers by limiting the presence of men in the wards [19, 21]. Therefore, the present study aimed to compare the KC by mothers with KC with the help of another female family member, namely the maternal grandmother, who culturally plays a significant role in the family during the birth of a newborn and her presence instead of the mother is entirely acceptable in the beliefs and culture of the studied country and the research setting. The objective of this clinical trial was to assess the effectiveness of KC by maternal grandmother, compared to KC by mother for hospitalized preterm newborns. Our hypothesis was that these methods of kangaroo care are not different in improving the stability of the newborns’ vital signs according to changes in their heart rate, respiration rate, SPO_2_, and body temperature.

## Methods

### Design and setting

This study was a randomized clinical trial with a parallel, pre-, and post-intervention design in which two methods of KC were evaluated in a single study. The study population consisted of the mothers or maternal grandmothers of all the preterm newborns who were admitted to the neonatal or NICU wards at Imam Khomeini Hospital in Kuhdasht, which is one of the hospitals affiliated with Lorestan University of Medical Sciences in Iran. The neonatal ward of this hospital has 6 incubators, and its NICU ward has 4 beds. In the year 2020, more than 250 newborns were admitted, of which 90 were premature neonates. Sometimes, due to the lack of beds and the desire of the parents, premature newborns are transferred to the hospitals in the center of the province.

### Participants, recruitment, and randomization

Considering alpha 0.05 and statistical power of 80%, based on the below formula and a similar study [16], 39 people were estimated for each group. Then, considering the possibility of dropping 20% of the sample, the sample size for each group was considered to be 47 people.

1 $$n=\frac{\left(Z_{1-\frac a2}+Z_{1-\beta}\right)^2(\delta_1^2+\delta_2^2)}{{{(\mu}_1-\mu_2)}^2}=\frac{\left(1.96+0.84\right)^2(11^2+{9.9}^2)}{{(6.6)}^2}=39$$

Participants were enrolled in the study by continuous non-probability sampling method of eligible samples according to the chronological order of neonates’ admission to the wards. The patients were divided into two groups. Allocation of patients to the groups was done using block randomization. Block randomization was used in order to balance the number of samples allocated to each of the studied groups. The block size was 4. In order to homogeneity the distribution of the confounders of gender (boy/girl), type of delivery (normal vaginal/cesarean section), birth weight (< 2000 g/ ≥ 2000–2500 g), and the newborns’ type of nutrition (mother’s breastfeeding/breast milk by other routes) based on these variables stratified block randomization was created and then samples randomly were placed the groups in a balanced way. The random sequence was created by the epidemiologist consultant using the block randomization package in the R software. For allocation concealment, sealed envelopes were used, which were kept by the secretary of the departments who were not a member of the research team. In order to preserve the random sequence, the outer surface of the envelopes was numbered in order. The lids of the letter envelopes were glued and placed in a box respectively. At the time of sampling, when a new patient was eligible to take part in the study, the researcher asked the secretary of the departments which group to assign the new participant. Subsequently, they were divided into two groups, including a group that received mother KC (control) and another group with maternal grandmother KC (intervention). The sampling period lasted from 15–1-2021 to 30–6-2021.

### Eligibility criteria

Premature newborns were included in the study if they were/had:

✓ 4–5 days old of age
✓ Gestational age between 32 and 36 weeks
✓ Birth body weight of 1500–2500 g
✓ The Apgar score between 7 and 10
✓ The conditions to be safely taken out of the incubator (SpO_2_ ≥ 85%, RR = 30–60/min, HR = 120–160/min)

The premature newborns not entering the study if:

✓ She/he had congenital abnormalities, neuro-muscular or skin disorders, a surgical wound on the chest, or had an umbilical vein catheter
✓ Her/him mother/maternal grandmother suffering from a physical disability, fever or infectious disease, wounds on the chest or abdomen, addiction, or mental problems such as psychosis or postpartum depression

The premature newborn was excluded from the study if:

✓ Her/him mother or maternal grandmother did not have a tendency to continue participating in the study, had physical or mental problems, or suffering from fever or infectious disease during KC
✓ The newborn’s need for surgery, IV catheter, receive or exchange blood, SpO_2_ < 85%, 30 ≥ RR ≥ 60, 100 ≥ HR ≥ 180, 37.5 < T < 36 °C while receiving KC

### Intervention and implementation

**Fig. 1:**
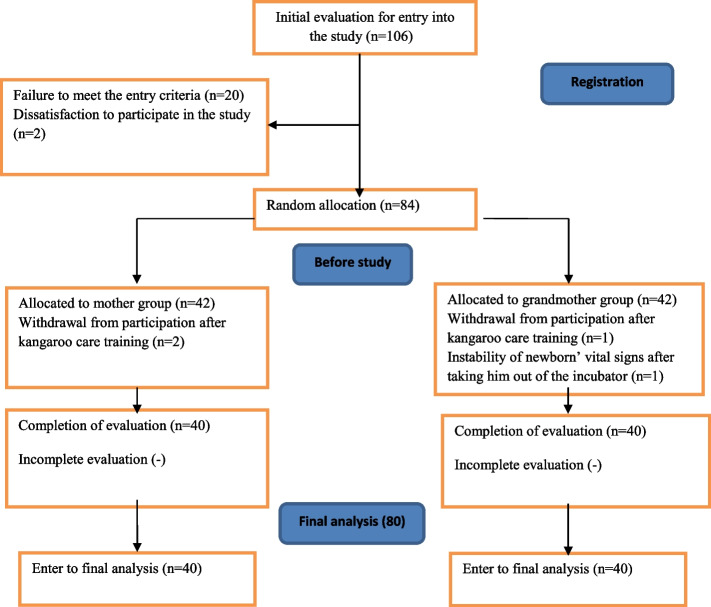
The study flowchart

One of the researchers and a trained research assistant first introduced themselves to the mother/maternal grandmother and briefed them on the study objectives. Then, they received individual training on KC using posters, images, and videos. The participants were also trained on personal hygiene, wearing special clothing, and not using fragrances, cigarettes, hookahs, or cellphones when providing KC. Then, prior to beginning the study, KC was performed twice separately by the caregivers under the supervision of the researcher. The diapered newborn was removed from the bed or incubator with the help of the researcher and placed upright on the caregiver’s chest so that the newborn’s head and chest rested on the caregiver’s chest and skin-to-skin contact was established. The newborn was covered with a sheet, and their head was covered with a hat to prevent a drop in their body temperature. The caregiver performed KC at a 30–60° angle while resting on a chair and leaning back slightly as they supported the newborn’s body and neck with their hands. To minimize measurement errors due to possible fluctuations in ambient temperature and physiological parameters throughout the day, the KC and the measurement and recording of these indicators were carried out for all newborns within the same time period from 15:00 to 17:00 on the fourth or fifth day of birth. KC was performed in a separate room (given the conditions at the neonatal unit of the study setting, including the small space and crowded ward) at temperatures of 26–29 °C. During the intervention, a warmer, oxygen, oxygen hood, and an incubator were available for newborns who might experience significant changes in vital signs during the study. In the case of instability of the newborn’s vital signs during the study (SpO_2_ < 85%, temperature < 36 °C, respiration rate > 60 and < 30/min, and heart rate of > 180 and < 100/min), they were removed from the study and immediately returned to the incubator. The flowchart for the study is shown in Fig. 1.

### Clinical outcome measures

The primary outcome of this study was the effect of maternal and maternal grandmother KC on vital signs and their comparison which were measured with the following tools. Due to the short duration of the intervention (because of the coronavirus restrictions) in this study, the secondary outcome was not investigated.

Data collection tools were a demographic form for the newborns (age, gender, type of feeding, birth method) and the mothers/maternal grandmothers’ demographic form for age, education, and job. The newborns’ vital signs were also recorded in the data collection form.

The newborns’ body temperature, heart rate, respiration rate, and SpO_2_ were assessed and recorded three times 15 min before the start of KC, 15 min after the start of care, and 15 min after the end of care. The newborns’ SpO_2_ level and heart rate were measured with a BCI 3180 pulse oximeter with a ± 0.3% error, and the device probe was taped to the right foot of the newborns. The newborns’ body temperature was measured over the forehead skin with a digital thermometer which had an error rate of ± 0.3 °C. The respiration rate was measured and recorded by the trained research assistant through direct observation for one min.

The validity of the pulse oximeter and thermometer, which had been selected from standard brands, was confirmed based on the manufacturer’s instructions. The pulse oximeter and digital thermometer were calibrated at the start of sampling. The reliability of the measurements and observation of the respiration rate was assessed simultaneously by two people. To determine the reliability of the respiration rate measurement in the first ten samples, two observers were recruited. To evaluate the inter-rater agreement, the intra-cluster correlation coefficient (ICC) was used, and the ICC was calculated as 0.97. Due to the high level of agreement, the rest of the respiration rate measurements were performed by only one observer.

### Data analysis

The data analysis was done by the statistical consultant, who was blind to the allocation of participants to the groups. Descriptive statistics were used to describe the data, mean and standard deviation for quantitative variables, frequency, and percentage for qualitative variables. In order to compare the groups, first, the normality of the data was assessed and confirmed using the Shapiro–Wilk test. Due to the normality of data distribution, the independent *t*-test and repeated measures ANOVA were used to analyze the data. Also, in order to compare qualitative variables in two research groups, the chi-square test and Fisher’s exact test were used. All statistical tests were performed using the SPSS version 22 software, and *P* < 0.05 was considered as the significant level.

## Results

Data from 80 newborns were analyzed; 40 were allocated to the mother KC group (control group) and 40 to the maternal grandmother KC (intervention group). There was no significant difference between the groups concerning newborns’ demographic variables (*P* > 0.05) (Table 1).

**Table 1:** The frequency, percentage, and homogeneity of the newborns to demographic variables in the mother kangaroo and grandmother kangaroo groups

**Variables**		Control		Intervention		*P* value*
		***N***	**%**	***N***	**%**	
**Infant’s age**	Day 4	9	22.50	6	15	0.568
	Day 5	31	77.50	34	85	
**Infant’s gender**	Boy	23	57.50	20	50	0.654
	Girl	17	42.50	20	50	
**Infant’s feeding**	Breast milk (breastfeeding)	1	2.50	–	–	1
	Breast milk (bottle)	39	97.50	40	100	
**Birth method**	Vaginal delivery	25	62.50	31	77.50	0.222
	Cesarean section	15	37.50	9	22.50	

^*^Chi-square test

The mean (standard deviation) age of mothers and maternal grandmothers was 29.27 (7.25) and 51.10 (8.80), respectively. The majority educational level of mothers (40%) was diploma, and maternal grandmothers (67.5%) were illiterate. The occupation of the majority of mothers (92.5%) and all of the maternal grandmothers was housekeeper.

There was not any significant difference in the newborns’ heart rate, respiration rate, SpO_2_, and body temperature between the groups based on the group effect (*P* > 0.05). There was a significant difference in the newborns’ heart rate, respiration rate, and SpO_2_ at the different stages of research based on the time effect (*P* < 0.001), but the difference was not significant for the newborns’ body temperature (*P* = 0.224). There was also a significant difference in the newborns’ heart rate, respiration rate, and SpO_2_ levels based on the group-time interaction effect (*P* < 0.05) (Table 2).

**Table 2:** Comparison of the newborns’ vital signs in different stages of the research in each group

**Variables**	Source	*F*	*P*-value*
**Infant’s heart rate**	Group	0.23	0.631
	Time	49.30	< 0.001
	Group × time	3.37	0.037
**Infant’s respiration rate**	Group	0.08	0.928
	Time	65.94	< 0.001
	Group × time	2.62	0.075
**Infant’s SpO**_2_	Group	0.42	0.516
	Time	107.34	< 0.001
	Group × time	1.59	0.206
**Infant’s body temperature**	Group	0.07	0.787
	Time	1.51	0.224
	Group × time	0.15	0.853

^*^Repeated measures ANOVA

Based on the results, there was no significant difference (*P* > 0.05) between the study groups in terms of the newborns’ heart rate, respiration rate, body temperature, and SpO_2_ level at different stages of the study (before, during, and after KC) (Table 3).

**Table 3:** The descriptive statistics and results of the independent *t*-test for the newborns’ vital signs in study groups

**Variables**	**Group**	**Before KC, mean (SD)**	**During KC, mean (SD)**	**After KC, mean (SD)**
**Heart rate**	Mothers	144.32 (9.53)	139.12 (8.05)	141.87 (8.30)
	Maternal grandmothers	145.60 (7.34)	137.63 (5.36)	139.95 (5.47)
	*P* value*	**0.505**	**0.330**	**0.225**
**Respiration rate**	Mothers	48.52(6.52)	44.25(5.65)	45.62(5.11)
	Maternal grandmothers	48.70 (4.93)	43.05 (5.32)	46.35 (4.53)
	*P* value*	**0.893**	**0.332**	**0.505**
**SpO**_2_	Mothers	94.30 (1.88)	96.47 (1.66)	95.90 (1.37)
	Maternal grandmothers	94.10 (1.15)	96.60 (1.31)	95.45 (1.23)
	*P* value*	**0.964**	**0.676**	**0.158**
**Body temperature**	Mothers	37.07 (0.22)	37.04 (0.09)	37.03 (0.10)
	Maternal grandmothers	37.07 (0.18)	37.05 (0.13)	37.04 (0.07)
	*P* value*	**0.876**	**0.502**	**0.430**

^*^Independent *t*-test

## Discussion

This study was conducted to compare the effect of KC by mothers and maternal grandmothers on the vital signs of preterm newborns. The findings showed that the newborns studied in this research were homogenous in terms of age, gender, feeding, and birth method without any significant differences between them. When the mother or maternal grandmother was providing KC, the newborns’ heart rate and respiration rate were lower compared to before and after receiving KC. On the other hand, the SpO_2_ level during KC was higher compared to before and after KC, while the temperature remained constant. These findings show that providing KC by both the mothers and the maternal grandmothers stabilized (improved) the newborn’s vital signs in equal measure.

In both the mother and maternal grandmother KC groups, the respiration rate decreased. Although there was an increase in this rate again after KC, it was still lower than the levels of it before KC. This effect was the same for KC given by the mother and maternal grandmother. In line with the findings of this research, the studies by Cho et al. and Gebuza et al. showed that KC can be effective in stabilizing newborns’ physiological performance, such as respiration rate [22, 23]. Moreover, during KC, oxytocin is released in the insular cortex of the brain, reducing stress and stabilizing the newborn’s vital signs [11, 24, 25]. Therefore, the provision of KC by either the mother or maternal grandmother is recommended for stabilizing respiration.

The findings also showed that the newborns’ heart rate dropped during KC in both the mother and maternal grandmother groups compared to before and after KC. Although the heart rate increased after KC, it was still lower than before the intervention. Deng et al. in their study protocol refer to the studies that stated skin-to-skin care in preterm newborns by another family member (father) leads to the better persistence of physiological parameters, including the stabilization of heart rate and neurological outcomes [14]. In research by Linnér et al., the heart rate in newborns under KC was more stable compared to newborns under routine care. The reduced heart rate during KC, whether given by the mother or maternal grandmother, may be a result of the newborns feeling calmer, upon which their autonomic activities and their heart rate and stress consequently will decrease [7]. Hence, the delivery of KC by either the mother or maternal grandmother is recommended for stabilizing newborns’ heart rates.

The results also showed that the newborns’ SpO_2_ was optimally increased during KC provided by the mother or maternal grandmother compared to before and after KC. This increase continued even after receiving KC compared to before. Other researches also showed that the SpO_2_ was significantly higher and more stable in newborns under KC than the newborns put in the incubator [8, 26]. The results reported by Bera et al. showed that the SpO_2_ levels increased during and after KC, so the newborns’ need for oxygen supplementation decreased 15 min after the start of KC [27]. There is a hypothesis that the increase and stability of SpO_2_ levels in newborns during KC may be due to the increased diaphragm efficiency and pulmonary function and improved oxygen delivery because of the vertical positioning of the newborn on the caregiver’s chest [28, 29]. Therefore, KC by both the mother and maternal grandmother is recommended for improving SpO_2_ in newborns.

The results further showed that the newborns’ body temperature during KC in both the mother and maternal grandmother did not differ significantly from before and after the newborns were put in an incubator. As it is warm in the incubator and preterm newborns have difficulty regulating their body temperature, they are expected to experience a drop in body temperature when taken out of the incubator. Similar studies showed that newborns receiving KC experience a significant improvement in problems such as hypothermia and hypoglycemia compared to newborns receiving routine care [26, 30, 31].

Since the newborn sensory system is highly sensitive, when they are embraced, newborns will sense heart rate, breathing, temperature, and body position, a sensory experience that helps stabilize temperature and heart rate and reduce respiratory rate during KC [22].

In a kangaroo position, the newborn is placed vertically over the mother’s chest, the limbs are bent to the frog position, and the head tilted to one side with the neck straight to facilitate breathing and avoid asphyxiation. This vertical position is better for oxygenation compared to sleeping on the back, increases lung volume, helps newborns achieve their movement milestones faster, and prevents asymmetric postures [29, 32]. It is therefore likely that the newborns’ position during KC in anybody’s arms, whether the mother or maternal grandmother, the establishment of skin-to-skin contact, the secretion of oxytocin, and the reduced autonomic system activity help maintain the newborns’ temperature and improvement other vital signs, such as reducing respiration rate and heart rate and increasing SpO_2_ levels.

## Conclusion

The findings showed that KC by maternal grandmother is as effective as KC by mother in stabilizing body temperature and improving other vital signs such as heart rate, respiration rate, and SpO_2_ levels in preterm newborns. Accordingly, if the mother is unable to be present at the hospital or provide KC to her newborn, another member of the family, such as the maternal grandmother, can help as a substitute for the mother.

### Strength and limitations

One of the strengths of this randomized clinical trial design is creating a positive attitude toward family-centered care in the health workers of neonatal departments. It shows that in conditions when the mother is not able to take care of the baby for any reason, the participation of another family member who is closely related to the mother in taking care of the baby can be helpful. KC is an effective strategy to increase family well-being, which can be done not only by the mother but also by other family members. Another strong point was the short time it takes to perform KC and its effect on the stabilization of the vital signs of premature infants.

In this study, due to the ethical considerations and presence of the mother in different situations and her taking part in the care of the premature neonate, the control group was the mother.

One of the limitations of this study is the small size of the sample. Due to the coronavirus pandemic, the parents of the newborns were willing to discharge their newborn from the hospital sooner for fear of contracting the coronavirus. This issue caused an increase in the sampling time. Also, during the corona pandemic, on the part of the hospital to reduce the transmission of the disease, there was a restriction to visits in the hospital, which did not let us assess the longer outcomes of KC. Therefore, it is suggested that more research be done on KC by a maternal grandmother over a longer period of time and more often to investigate secondary outcomes of KC such as weight gain, length of hospitalization, rate of infection, quality of sleep, attachment behavior, and feeding of the newborn.

Also, in the present study, KC was performed once during the day and for 30 min; it is suggested future researches focus on possible long-term benefits of KC on mothers and newborns.

It is also suggested to compare the effect of providing KC by another family member, especially the sibling of the newborn, or the paternal mother, aunt, uncle, and grandparents, or even by the medical staff as caregivers with KC by the mother.

Also, KC by maternal grandmother and her presence in the hospital, who had a close relationship with her mother, can make the mother calmer, helped, and satisfied. So, it is suggested to assess the effect of KC by the maternal grandmothers on the stress, anxiety, depression, self-confidence, and quality of life of the mothers.

## Data Availability

The data set used during the study is available to the authors.
